# Enhancing Care Coordination and Patient Engagement Through Electronic Medical Record Utilization in Primary Healthcare: A Mixed-Methods Study

**DOI:** 10.3390/healthcare14111458

**Published:** 2026-05-25

**Authors:** Sarah Mareta Devira, Ferdi Antonio, Deffina Widjanarko

**Affiliations:** Department of Hospital Administration, Pelita Harapan University, South Jakarta City 12930, Indonesia; sarahdevira@gmail.com (S.M.D.); deffina.ruth@gmail.com (D.W.)

**Keywords:** electronic medical records, primary healthcare, EMR utilization, care coordination, patient engagement, healthcare delivery, interprofessional collaboration

## Abstract

**Highlights:**

**What are the main findings?**

- EMR utilization in primary healthcare is primarily driven by clinical workflow alignment and digital health competency, beyond system availability alone.
- Interprofessional collaboration and patient engagement in community health centers act as key mechanisms linking system capability to effective EMR use.

**What are the implications of the main findings?**

- Improving EMR utilization can strengthen care coordination, enhance patient engagement, and increase service efficiency in primary healthcare settings.
- Digital transformation in community health centers efforts should prioritize workflow integration, user competency development, and balanced governance to support sustainable system use.

**Abstract:**

Background: Primary healthcare systems continue to face patient safety challenges, particularly misdiagnosis and medication errors, which contribute to preventable harm and reduced quality of care. Electronic Medical Records (EMRs) have the potential to improve clinical documentation, support decision-making, and reduce risks; however, these benefits depend on effective utilization in routine clinical practice. This study examined factors influencing EMR utilization in primary healthcare settings. Methods: A sequential explanatory mixed-methods design was conducted across 42 community health centers in one Indonesian city. Quantitative data from general practitioners were analyzed using Partial Least Squares Structural Equation Modeling (PLS-SEM) to examine the relationships among clinical workflow fit, digital health competency, governance, system capabilities, interprofessional collaboration, perceived patient engagement, and EMR utilization. Qualitative interviews were subsequently conducted to provide a contextual explanation of the quantitative findings. Results: Clinical workflow fit and digital health competency emerged as the strongest factors associated with EMR utilization. Their effects operated through interprofessional collaboration and perceived patient engagement, indicating the importance of integrating EMRs into everyday clinical workflows. Governance structures and system capabilities primarily functioned as enabling conditions rather than direct determinants of utilization. Qualitative findings further highlighted the importance of practical workflow integration, communication processes, and user competency in supporting meaningful system use. Conclusions: EMR utilization may contribute to improved care coordination, patient engagement, and service efficiency in primary healthcare settings. Strengthening workflow alignment and digital competency may help support safer and more reliable care delivery, particularly in resource-constrained environments where risks of misdiagnosis and medication errors remain significant.

## 1. Introduction

Digital transformation has become a central agenda in health systems worldwide, as governments and healthcare organizations increasingly invest in digital infrastructures to improve service quality, enhance patient safety, and support data-driven decision-making [1,2]. Among these technologies, Electronic Medical Records (EMRs) play a critical role as core health information systems that support clinical documentation, care coordination, and integrated patient management [3,4].

Primary healthcare services represent the frontline of health systems; however, they continue to face persistent challenges in patient safety, particularly misdiagnosis and medication errors. Globally, diagnostic errors in primary care are among the leading causes of preventable harm, contributing to increased morbidity, rising healthcare costs, and declining patient trust [5]. These risks are further intensified in urban settings characterized by high patient volumes, limited consultation time, and increasingly complex clinical conditions. In the Indonesian context, primary healthcare services face similar systemic pressures due to resource constraints and variability in service quality, which may compromise patient safety, therapeutic effectiveness, and continuity of care [6].

In response to these challenges, digital health technologies have been increasingly adopted as potential solutions to improve patient safety and care quality. EMR systems, in particular, are widely recognized for their potential to reduce clinical errors through integrated documentation, real-time access to patient information, and data-informed decision-making [7,8]. While EMRs offer significant promise, the extent to which these benefits are realized in practice depends heavily on how consistently and effectively they are used in routine clinical workflows [9,10].

Previous studies have extensively examined the relationship between health information technology and patient safety, including the role of EMR in reducing medication errors and improving the quality of clinical documentation [11,12]. However, most of this evidence has been derived from secondary and tertiary care settings, while empirical findings in primary healthcare—particularly in middle-income countries with resource limitations—remain relatively scarce. These limitations are especially relevant in settings where variations in infrastructure, training, and organizational support lead to heterogeneous patterns of EMR use [13,14].

Beyond availability, EMR utilization is shaped by how well digital systems align with clinical workflows and are embedded in organizational processes. Clinical workflow fit (CWF), data governance maturity (DGM), and health IT affordances are key factors that influence how healthcare professionals use EMR systems and coordinate care [15,16]. These conditions interact with relational processes such as interprofessional collaboration (ICE), which supports care coordination, and perceived patient engagement (PPE), which reflects how digital systems facilitate patient-centered care. At the same time, EMR mastery level (EML) and technostress (TCS) act as important boundary conditions that may either enhance or constrain effective system use [17,18,19].

Despite increasing recognition of these multidimensional influences, current explanations of EMR utilization remain limited. Much of the existing literature has focused on technology adoption models, emphasizing perceived usefulness and ease of use as primary determinants [20]. However, such approaches provide limited insight into how EMR systems contribute to improvements in care processes and service delivery. As highlighted by Holden and Karsh [21], health information technology implementation is shaped by dynamic interactions between technology, tasks, users, and organizational context. Understanding EMR utilization, therefore, requires a broader perspective that captures how these elements interact in real-world healthcare settings.

In this context, effective EMR utilization can be understood as a critical pathway through which digital technologies contribute to safer and more coordinated care. When EMR systems align with clinical workflows and are supported by user capabilities, they can enhance communication, improve coordination, and strengthen clinical decision-making [22,23]. Conversely, misalignment between system design and clinical practice may lead to workarounds, partial system use, and reduced efficiency, ultimately limiting the potential benefits of digital health systems [24].

These challenges are particularly relevant in the Indonesian primary healthcare context, where digital health initiatives are expanding rapidly, but implementation capacity remains uneven across facilities. Variations in infrastructure, training, and organizational support result in diverse patterns of EMR use, ranging from routine integration to fragmented or minimal utilization [14]. Understanding the factors that enable effective EMR utilization in such settings is therefore essential for improving service quality, strengthening care coordination, and enhancing patient engagement.

To address this gap, this study extends current understanding of EMR use by moving beyond adoption-based explanations toward a care-oriented perspective that emphasizes how digital systems support real-world clinical practice. Specifically, this study integrates clinical workflow alignment, user capability, and organizational conditions within a unified framework to explain how EMR utilization contributes to care coordination and patient engagement. By combining quantitative modeling with qualitative insights, this study provides a context-sensitive explanation of how EMR systems can be more effectively utilized to support healthcare quality in resource-constrained primary care settings.

## 2. Methods

### 2.1. Quantitative Phase

This study employed a quantitative explanatory design, using a cross-sectional survey, to examine the determinants of Electronic Medical Record use in Indonesian primary healthcare. The study population comprised general practitioners working at all 42 community health centers (Pusat Kesehatan Masyarakat or Puskesmas) in Palembang, Indonesia. As one of the urban areas in Indonesia, Palembang has a population of over five million inhabitants. Palembang has experienced ongoing digital transformation in healthcare services, including the adoption of EMR systems. The community health centers in Palembang serve a diverse patient population, mainly middle- to lower-class, making them relevant for examining healthcare workers’ experiences, perceptions, and acceptance of EMR implementation in routine clinical practice.

These primary healthcare facilities had implemented Electronic Medical Record (EMR) systems for at least 1 year before the study to ensure sufficient user experience with them. Eligible respondents included all physicians. The minimum required sample size was calculated using G*Power^®^3.1.9.7 analysis to ensure adequate statistical power for PLS-SEM estimation. The calculation with alpha 0.05 and power 80% with effect size 0.15 indicated a minimum sample requirement of 109 respondents, while this study included 217 respondents, exceeding the recommended threshold for model testing and hypothesis evaluation. Data were collected using a structured questionnaire distributed electronically through a secure online platform. Participation was voluntary and anonymous, and informed consent was obtained before survey completion [9].

**Figure 1:**
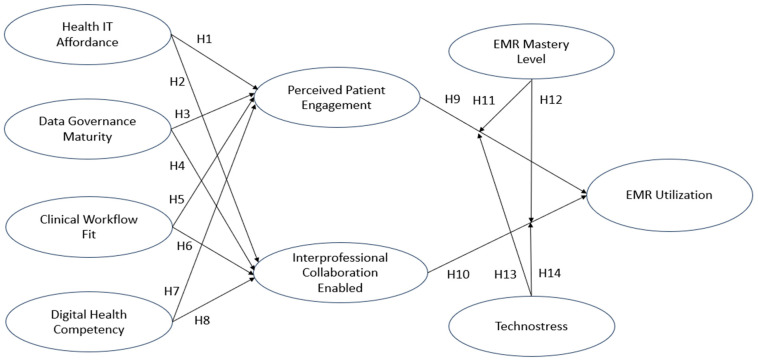
Conceptual framework.

Figure 1 presents the conceptual framework of the study, integrating organizational, individual, and technological dimensions to explain how EMR utilization supports healthcare delivery in primary care settings. The model positions health IT affordances (HIA), clinical workflow fit (CWF), data governance maturity (DGM), digital health competency (DHC), EMR mastery level (EML), and technostress (TCS) as key antecedents influencing interprofessional collaboration (ICE) and perceived patient engagement (PPE), which in turn shape EMR utilization (EMU).

The framework emphasizes that effective EMR use does not arise solely from system availability, but from the alignment between digital systems, clinical workflows, and user capability. Through these mechanisms, EMR utilization serves as a pathway to improve care coordination, enhance patient engagement, and increase the efficiency of primary healthcare delivery.

All constructs in this study are defined to reflect their role in supporting healthcare delivery and clinical practice. As outlined in Table 1, prior evidence shows that digital health systems with strong Health IT affordances, such as accessible information, interactive features, and self-management support, can promote more active patient involvement in healthcare services [25,26,27]. Additionally, higher levels of digital health maturity, especially robust data governance practices, support the continuity of patient records and the systematic use of patient-related information, thereby improving the delivery of coordinated, patient-centered care [28]. Beyond technological capabilities, the compatibility of digital health systems with existing clinical workflows also plays a key role, as systems that integrate smoothly into routine clinical practice are more likely to be adopted consistently, enabling more efficient communication and greater patient participation in care processes [29]. Furthermore, patient engagement in digital health settings is influenced by healthcare providers’ digital health competency, with higher competency allowing providers to use digital platforms more effectively for communication, patient education, and shared decision-making [30]. Overall, these findings indicate that technological, organizational, and individual factors collectively shape perceived patient engagement.

**H1:**  *Health IT affordance has a positive relation with perceived patient engagement*

**H3:**  *Data governance maturity has a positive relation with perceived patient engagement*

**H5:**  *Clinical workflow fit has a positive relation with perceived patient engagement*

**H7:**  *Digital health competency has a positive relation with perceived patient engagement*

The implementation of patient-accessible EMRs has been shown to influence healthcare delivery by enhancing transparency and patient empowerment, as well as improving information sharing among healthcare professionals. When clinical data are readily accessible and consistently documented, healthcare providers are better able to coordinate care, align clinical decisions, and communicate effectively across professional boundaries. Recent evidence suggests that such systems facilitate more integrated care processes by enabling timely access to patient information, which is essential for interprofessional collaboration [38,39,40].

In addition, an organization’s level of digital health maturity, particularly in data governance, plays a critical role in ensuring data accuracy, interoperability, and the continuity of patient records [41]. These capabilities support more structured communication and reduce fragmentation in care delivery, thereby strengthening collaboration among healthcare providers. Furthermore, the effectiveness of digital health systems in enabling collaboration depends on their alignment with clinical workflows, as systems that fit well within routine practice are more likely to support coordinated activities across professional roles [42].

At the individual level, digital health competency also contributes to interprofessional collaboration. In particular, healthcare providers with higher competency are better able to utilize digital platforms for information exchange, care coordination, and collaborative decision-making. Taken together, these organizational and individual capabilities illustrate how technological, organizational, and personal factors jointly enable interprofessional collaboration in digital health environments [28,29].

**H2:**  *Health IT affordance has a positive relation with interprofessional collaboration enabled*

**H4:**  *Data governance maturity has a positive relation with interprofessional collaboration enabled*

**H6:**  *Clinical workflow fit has a positive relation with interprofessional collaboration enabled*

**H8:**  *Digital health competency has a positive relation with interprofessional collaboration enabled*

Prior empirical evidence suggests that EMR systems are used more actively when they facilitate patient engagement. This includes improved communication, support for self-management, and greater patient involvement in clinical decision-making. When physicians see that EMR use enhances patient engagement, they are more likely to use the system consistently in clinical practice to support patient-centered care [26,27,30].

In addition to patient-related mechanisms, interprofessional collaboration also influences EMR use. EMR systems act as a centralized, accessible source of patient information. They enhance communication, coordination, and role clarity among physicians, nurses, and other healthcare workers [15,16,43]. Better coordination reduces information fragmentation and supports efficient teamwork. This consistency makes EMR use more meaningful in clinical workflows.

**H9:**  *Perceived patient engagement has a positive relation with EMR utilization*

**H10:**  *Interprofessional collaboration enabled has a positive relation with EMR utilization*

The integration of patient-centered digital health records has been identified as a transformative factor in modern healthcare, significantly influencing engagement levels and health outcomes [26,27]. Systematic evidence suggests that when patients are granted access to their electronic records, it creates a more transparent environment that directly correlates with increased healthcare engagement and improved longitudinal care. However, the success of these platforms is not solely dependent on technical availability; it is driven by a complex set of factors, including platform usability and the perceived value of digital health data to the patient [30]. In addition to patient-side factors, the effectiveness of EMR systems in primary care is deeply rooted in their ability to facilitate interprofessional collaboration. Research indicates that while EMR systems serve as a foundation for teamwork, their success depends on identifying specific facilitators, such as system interoperability, and mitigating barriers, such as high administrative burdens [15]. High-quality interprofessional collaboration in primary care, supported by digital tools, has been shown to enhance the overall quality of health services [16].

**H11:**  *EMR mastery level moderates the relationship between perceived patient engagement and EMR utilization*

**H12:**  *EMR mastery level moderates the relationship between interprofessional collaboration enabled and EMR utilization*

A nuanced understanding of the socio-technical environment is essential to address the organizational and individual pressures associated with digital health systems. In this context, technostress may act as a moderating factor, shaping how interprofessional collaboration and patient engagement translate into EMR utilization, particularly when increased digital demands create cognitive and operational burdens for healthcare professionals [15].

**H13:**  *Technostress moderates the relationship between perceived patient engagement and EMR utilization*

**H14:**  *Technostress moderates the relationship between interprofessional collaboration enabled and EMR utilization*

In the following steps, the measuring tool was created using verified instruments from earlier information systems and health informatics research and modified for use in primary care settings. The items were revised to fit the context of EMR use in primary healthcare in Indonesia after adaptation from previously validated measures and translated into Bahasa Indonesia using a contextual adaptation approach. The translated version was reviewed by experts to ensure semantic clarity, contextual relevance, and conceptual consistency. Minor wording adjustments were subsequently made after the face validity evaluation by three expert panels. A five-point Likert scale ranging from strongly disagree (1) to strongly agree (5) was used to measure each issue. A preliminary evaluation was also conducted to ensure that the items were understandable to the target respondents.

Before statistical analysis, the dataset was screened to ensure completeness, eligibility, and response quality. The Google Form questionnaire was designed using mandatory response settings to minimize missing data. Respondents who had never used an electronic medical record (EMR) system were excluded based on the predefined exclusion criteria. In addition, responses showing uniform answer patterns across all questionnaire items were reviewed and excluded to reduce the likelihood of non-differentiated or low-quality responses.

Partial Least Squares Structural Equation Modeling with SmartPLS^®^4.1.1.8 software was used to analyse the data. PLS-SEM was selected since the study aimed to examine complex relationships among multiple latent constructs, including mediating and moderating effects, while accommodating prediction-oriented analysis and non-normal data distribution characteristics. In addition, PLS-SEM is appropriate for exploratory studies in healthcare and information systems research involving relatively moderate sample sizes [44]. The measurement model evaluation included assessments of indicator reliability, internal consistency reliability, convergent validity, and discriminant validity. Indicator reliability was evaluated using outer loading values, while internal consistency reliability was assessed through Composite Reliability (CR) and Cronbach’s alpha values. Convergent validity was confirmed using the average variance extracted (AVE) threshold [44].

To minimize potential response bias in this survey study, several procedural remedies were implemented during the questionnaire design and data collection process. First, the survey was conducted anonymously, and no personally identifiable information was collected from participants. This approach was intended to reduce social desirability bias and encourage respondents to provide honest and unbiased answers. Second, the order of questionnaire items was randomized to minimize common method bias and reduce the likelihood of patterned or systematic responses caused by item sequencing effects. In the analyses step, to minimize the potential influence of common method bias (CMB), a full collinearity assessment approach was conducted using variance inflation factor (VIF) values. VIF values below the recommended threshold indicated that common method bias was unlikely to threaten the validity of the findings [45].

Discriminant validity was examined using the heterotrait–monotrait ratio (HTMT), with threshold values [46]. Furthermore, out-of-sample predictive power was assessed using the Cross-Validated Predictive Ability Test (CVPAT), which has been recommended for predictive model assessment in PLS-SEM studies [47]. In addition, Importance–Performance Map Analysis (IPMA) was conducted to extend the interpretation of the PLS-SEM results by simultaneously evaluating the importance and performance of constructs and indicators related to EMR utilization. IPMA helps identify areas with high importance but relatively lower performance that may represent potential priorities for improvement in EMR implementation and primary healthcare practice [48].

### 2.2. Qualitative Phase

The qualitative phase was conducted as part of a sequential explanatory mixed-methods design, in which qualitative inquiry was intentionally guided by the quantitative results from Phase 1. Specifically, statistically strong, non-significant, and moderating relationships were prioritized to provide explanatory depth and contextual understanding of the quantitative findings [49]. This approach enabled the qualitative phase to explore why particular statistical patterns emerged in practice, including the strong influence of digital health competency on perceived patient engagement and the moderating roles of EMR mastery level and technostress in EMR utilization. Explanatory sequential mixed-methods designs are particularly valuable for interpreting, contextualizing, and extending quantitative findings through in-depth qualitative inquiry [50,51].

Participants for the qualitative phase were purposively selected from respondents who completed the quantitative survey in Phase 1. Selection was based on variation in several key quantitative constructs, including digital health competency, technostress, and EMR mastery level, to ensure representation of diverse experiences related to EMR utilization in primary healthcare practice. The qualitative participants included general practitioners and heads of community health centers to capture both clinical and managerial perspectives on EMR implementation and utilization. Participants with relatively high and low construct scores were included to capture contrasting perspectives and implementation experiences. Qualitative data were collected through structured open-ended electronic interviews conducted iteratively until sufficient thematic depth and consistency of responses were achieved. The qualitative phase was specifically designed to help explain and contextualize the quantitative findings within routine clinical and organizational settings.

Theoretical saturation was assessed continuously throughout the qualitative phase. Recruitment and analysis proceeded concurrently until no substantial new codes, conceptual categories, or explanatory insights emerged from subsequent participant responses. Saturation was determined through repeated thematic redundancy observed across interviews and was collaboratively evaluated by the research team during iterative coding discussions. The final interviews largely confirmed previously identified themes rather than generating new conceptual findings, indicating sufficient explanatory depth had been achieved [52,53].

Qualitative data were analysed using a thematic analysis approach deductively informed by the quantitative findings [54]. Initial coding was guided by key quantitative relationships while remaining open to emergent contextual insights from participant narratives. Through iterative comparison, categorization, and theme refinement, explanatory themes were developed to clarify the underlying mechanisms influencing EMR utilization in primary healthcare settings. To strengthen methodological rigor, investigator triangulation was applied through independent coding, consensus discussions, and maintenance of an audit trail documenting analytical decisions. Integration between quantitative and qualitative strands occurred at the interpretation stage using joint display matrices to facilitate meta-inference development and mixed-methods integration [55,56]. This integration generated a more comprehensive understanding of how digital health competency, EMR mastery level, workflow alignment, and technostress interact to shape EMR utilization in real-world primary healthcare practice.

## 3. Results

### 3.1. Respondent Profile

Out of 230 online questionnaires distributed during the data collection period, 217 responses were valid after completeness and eligibility screening, and therefore retained for data analysis, resulting in a valid response rate of 90%. Responses with incomplete submission, failure to meet screening criteria, or straight-lining patterns were excluded. The final sample comprised 217 general practitioners from 42 community health centers in Palembang, Indonesia. The demographic profiles are summarized in Table 2.

Table 2 presents the demographic and professional characteristics of the respondents *(n* = 217). All participants held an active medical license (100%). The majority were civil servants (51.6%), followed by government contract employees (24.9%) and other employment statuses (23.5%). All respondents had experience using Electronic Medical Records (EMRs) (100%). In terms of sex, 73.3% were female, and 26.7% were male. The largest age group was 31–40 years (43.8%), followed by 20–30 years (35.5%) and 41–50 years (18.4%). Regarding education, most respondents held a Bachelor of Medicine/Professional degree (69.1%), while smaller proportions had a Master’s degree (18.4%) or specialist qualification (8.3%). Regarding work experience at the community health center, 25.8% had worked for 1–3 years, and 16.1% had more than 10 years of experience. Additionally, 46.6% had received EMR training, indicating that the majority of respondents (53.5%) were familiar with digital health systems in their workplace.

### 3.2. Measurement Model

#### 3.2.1. Indicator Reliability and Validity

Indicator reliability was assessed using outer loadings from SmartPLS^®^ analyses. As shown in Table 3, all indicators across the constructs demonstrate loadings above the recommended threshold of 0.70. Several indicators were less than 0.7, but higher than 0.4, which can occur in PLS-SEM but did not invalidate the model, since the AVE and Cronbach alpha values were still accepted. These results confirm that all indicators adequately represent their respective latent constructs.

These findings indicate that indicator reliability is satisfied. Almost all indicators across the constructs CWF, DGM, DHC, EML, EMU, HIA, ICE, PPE, and TCS exhibit outer loadings that are generally above the minimum threshold of 0.70. This indicates that each indicator has a strong capacity to represent its respective latent construct. Overall, these findings confirm that indicator reliability has been achieved and that all indicators are appropriate for retention in the research model. Internal consistency was evaluated using Cronbach’s alpha and Composite Reliability. Convergent validity was assessed using average variance extracted. As shown in Table 3, all constructs exceed recommended thresholds of 0.60 for Cronbach’s alpha, 0.70 for Composite Reliability, and 0.50 for AVE.

As a result, the measurement model exhibits convergent validity and good internal consistency. All constructs attain Cronbach’s alpha values of 0.60 or higher, indicating adequate internal reliability (see Table 3). Additionally, all constructs’ Composite Reliability (rho_c) values are higher than the suggested cutoff of 0.70, indicating good internal consistency across the latent variables. Each construct’s average variance extracted (AVE) values are also more than 0.50, indicating that each construct accounts for more than half of the variation in the indicators. Thus, the measurement model satisfies the conditions for both convergent validity and reliability.

#### 3.2.2. Discriminant Validity

Discriminant validity was evaluated using HTMT. All values fall below 0.90, indicating acceptable discriminant validity. Selected results are shown in Table 4.

Discriminant validity was assessed to ensure that the factors in this research are distinct from one another, as shown in Table 4. The HTMT ratios for all factors were below the 0.90 threshold. To examine this relationship, HTMT bootstrapping intervals were used. Discriminant validity is established when the 95% confidence interval does not include 1.00. This requirement is met, meaning that, although highly interrelated, these two factors are still distinct from a statistical perspective, thereby satisfying the discriminant validity criterion.

### 3.3. Structural Model

The structural model explains a substantial proportion of variance in the key endogenous constructs. EMR utilization demonstrates a relatively strong explanatory power (R^2^ = 0.714), indicating that the proposed model captures the main drivers of system use in primary care settings. Interprofessional collaboration (R^2^ = 0.536) and perceived patient engagement (R^2^ = 0.479) are also moderately explained, suggesting that both organizational and individual-level factors play meaningful roles in shaping these outcomes.

**Figure 2:**
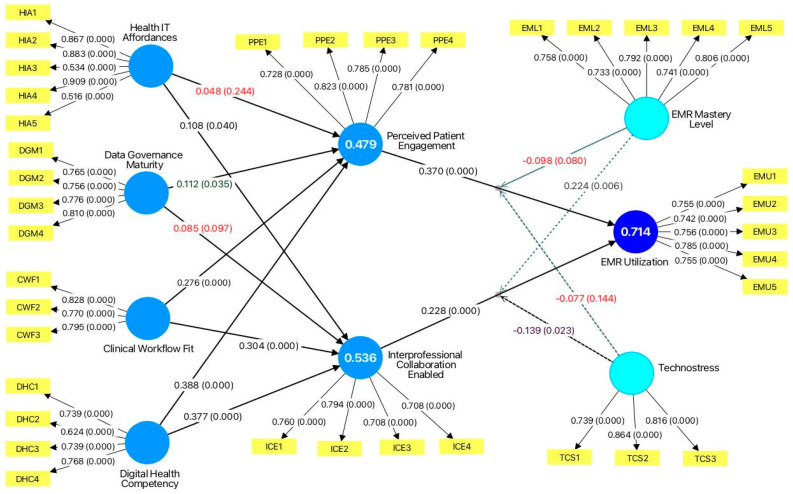
Inner model.

Figure 2 above illustrates the outcome of the PLS-SEM structural model, including path coefficients and the direction of relationships among constructs. The diagram shows that digital health competency and workflow fit exert strong positive effects on ICE and PPE, which subsequently have significant impacts on EMR use. The figure also visualizes moderation effects involving EMR mastery level and technostress support on selected relationships toward EMU. The magnitude of the path coefficients highlights the relative strength of each relationship, making Figure 2 a comprehensive visual summary of the hypothesis testing, mediation, and moderation results presented in the statistical tables.

#### 3.3.1. Predictive Validity

Predictive assessment using CVPAT (Cross-Validated Predictive Ability Test) shown in Table 5, confirms strong predictive capability, as PLS loss values are consistently lower than IA loss values with significant *p*-values.

The CVPAT results presented in the table indicate that the PLS loss values are lower than the IA loss values for the ICE, PPE, and EMU constructs, as well as for the overall model. The negative, statistically significant average loss difference (*p*-value < 0.05) indicates that the PLS model outperforms the benchmark model. Therefore, it can be concluded that the structural model exhibits strong predictive validity and is appropriate for predictive purposes.

#### 3.3.2. Hypothesis Testing

The following Table 6 presents the results of hypothesis testing using the Partial Least Squares Structural Equation Modeling (PLS-SEM) approach. The significance of the structural relationships was evaluated based on confidence interval and *p*-value obtained through the bootstrapping procedure.

The hypothesis testing results indicate that 10 out of the 14 proposed hypotheses were supported, while four hypotheses were not supported. Among the supported relationships, the strongest direct effects were observed for digital health competency on perceived patient engagement (β = 0.388, *p* < 0.001) and interprofessional collaboration enabled (β = 0.377, *p* < 0.001), highlighting digital competency as the most influential determinant in the model. Clinical workflow fit also demonstrated significant positive effects on interprofessional collaboration enabled (β = 0.304, *p* < 0.001) and perceived patient engagement (β = 0.276, *p* < 0.001).

Furthermore, both perceived patient engagement (β = 0.370, *p* < 0.001) and interprofessional collaboration enabled (β = 0.228, *p* < 0.001) significantly enhanced EMR utilization. In regard to moderating effects, EMR mastery level significantly strengthened the relationship between interprofessional collaboration enabled and EMR utilization (β = 0.224, *p* = 0.006), whereas technostress significantly weakened the relationship between perceived patient engagement and EMR utilization (β = –0.139, *p* = 0.023). In contrast, four hypotheses were not supported, namely the effects of health IT affordances on perceived patient engagement (H2), data governance maturity on interprofessional collaboration enabled (H3), EMR mastery level on the relationship between perceived patient engagement and EMR utilization (H12), and technostress on the relationship between interprofessional collaboration enabled and EMR utilization (H13), as their *p*-values exceeded the 0.05 significance threshold.

The results point to health IT affordances as a relevant starting point in the model, although their influence does not unfold in the same way across all relationships. In practice, these affordances appear to matter more for how professionals coordinate with one another than for how patients become engaged. The link between interprofessional collaboration and perceived patient engagement is clear and statistically supported, yet when it comes to perceived patient engagement, the effect seems to fade, or at least become less convincing.

From the perspective of the capability-related factors, a slightly different pattern emerges. Clinical workflow fit and digital health competency consistently show strong and positive associations with both collaboration and patient engagement. That consistency is hard to ignore. When EMR features align with daily clinical routines, such as documenting anamnesis, ordering lab tests, or managing chronic patients in programs, and when clinicians feel confident navigating those features, both teamwork and patient involvement tend to improve in a fairly predictable way. Data governance maturity, however, behaves a bit differently. Its contribution is visible on the patient side, particularly in ensuring data accuracy, confidentiality, and structured documentation, but it does not seem to extend as clearly into interprofessional interactions.

Further down the model, both interprofessional collaboration and perceived patient engagement show meaningful positive effects on EMR utilization. This suggests that EMR use is not driven purely by system availability or technical design, but also by how people interact around it. In other words, when communication flows well between providers and patients are more actively involved, the system is more likely to be used consistently and effectively. These two constructs seem to function as practical bridges, translating upstream organizational and individual factors into actual system use.

The moderation findings add another layer, though not uniformly. EMR mastery level strengthens the relationship between collaboration and EMR utilization, which makes intuitive sense. When users are more proficient, collaborative efforts are more easily translated into meaningful system use. On the other hand, the expected interaction between mastery and patient engagement does not reach statistical significance, suggesting that proficiency alone may not be enough to amplify patient-driven dynamics.

A similar selective pattern appears with technostress. Its moderating role is evident in the relationship between patient engagement and EMR utilization, indicating that higher levels of stress related to technology can shift how engagement translates into actual use. In contrast, its influence on the collaboration pathway is not statistically supported, which is somewhat surprising and perhaps worth further exploration.

Comprehensively, these findings suggest that moderation in this model does not operate in a blanket fashion. Instead, it seems to intervene in specific relational pathways, at times strengthening and at other times leaving relationships unchanged. This selective behavior highlights the need to look beyond direct effects and consider how contextual factors, such as user mastery and technological strain, shape the way EMR systems are ultimately used in primary care settings.

#### 3.3.3. Mediation Analysis

Mediation effects are selective, with stronger mediation observed for workflow fit and digital competency. The results presented in Table 7 indicate that ICE and PPE function as mediators in several relationships between exogenous variables and EMU. The indirect effects of CWF and DHC on EMU via both ICE and PPE are statistically significant, suggesting strong mediation mechanisms. In contrast, the indirect effect of HIA on EMU through either ICE or PPE is not significant. DGM demonstrates a significant indirect effect through PPE, but not through ICE. These findings suggest that the mediating roles of ICE and PPE are selective, depending on the specific antecedent variables that influence them.

#### 3.3.4. Moderation Analysis

The moderating effects were examined using simple slope analysis, where conditional effects were plotted at the mean and at one standard deviation above and below the mean values. Among the four tested moderation relationships, two demonstrated meaningful interaction patterns.

**Figure 3:**
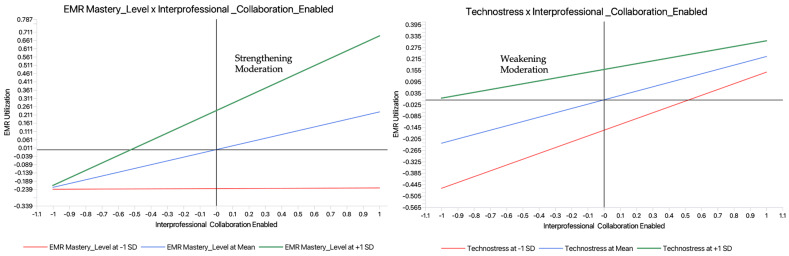
Simple slope moderation.

The results as visualized in Figure 3 indicate that EMR mastery level positively moderates the relationship between interprofessional collaboration and EMR utilization. Higher levels of mastery were associated with a stronger positive relationship between collaboration and system utilization. In contrast, technostress demonstrated a negative moderating effect, weakening the relationship between interprofessional collaboration and EMR utilization, particularly at lower levels of collaboration.

Overall, the findings suggest that EMR mastery level and technostress may influence how collaborative processes relate to EMR utilization in primary healthcare settings. These results highlight the importance of user competency and the potential impact of technological strain on effective system use.

### 3.4. Importance–Performance Map Analysis (IPMA)

The Importance–Performance Map Analysis (IPMA) was conducted to extend the PLS-SEM results by simultaneously evaluating the importance (total effects) and performance (average latent variable scores) of each construct in explaining EMR use.

**Figure 4:**
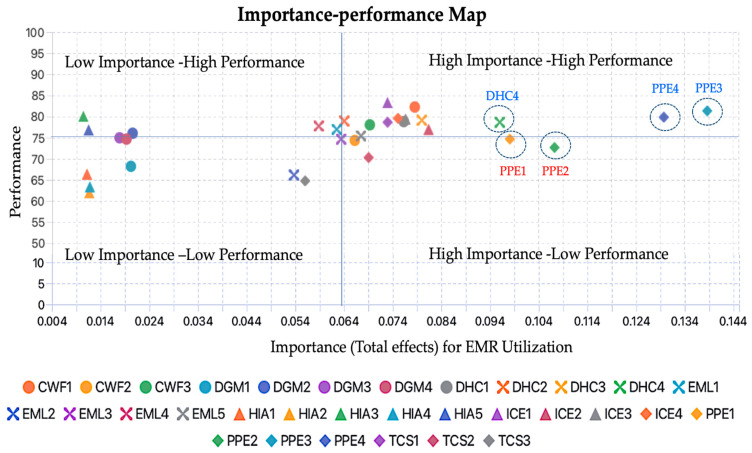
IPMA indicator.

At a more detailed level, the Importance–Performance Map Analysis (IPMA) helps move the discussion beyond overall constructs and into the specific items that appear most influential in relation to EMR utilization. The IPMA result, as depicted in Figure 4, helps identify areas that could benefit from additional attention during implementation improvement efforts, and can benefit the improvement process. Items that fall into the high-importance but still moderate-performance range are particularly revealing. They matter a great deal for EMR use, yet in practice, they are not performing as well as they could.

A closer look at the results points to several areas that feel quite familiar in everyday clinical work. Indicators related to workflow efficiency, such as how quickly patient histories can be accessed or how smoothly documentation follows the consultation process, merged as relatively important within the IPMA findings. The same is true for elements tied to interprofessional communication, especially when clinicians need to coordinate care across units. Within the model, certain indicators under digital health competency, including DHC4, and patient engagement measures like PPE1 and PPE2, appear in that high-importance but not fully optimized zone. This suggests that even small, targeted improvements, perhaps additional training on specific EMR features or clearer prompts during patient interaction, could potentially support improvements in EMR utilization.

On the other hand, not all areas require immediate intervention. Some indicators, such as PPE3 and PPE4, already show both high importance and strong performance. These aspects seem to be functioning as intended, maybe reflecting relatively consistent implementation practices among clinicians, such as explaining treatment plans or involving patients in follow-up visits. Maintaining these strengths is just as important as improving weaker areas.

The research finding of the pattern suggests that practical, system-level refinements may represent feasible areas for incremental improvement. Adjustments that make the interface more intuitive, reduce redundant data entry, or support clearer communication between providers may help support more effective day-to-day EMR utilization on how EMRs are used day to day. In that sense, the IPMA complements the structural model by providing additional practical interpretation at the indicator level. It translates statistical relationships into concrete priorities, offering a clearer direction for improving EMR implementation in primary care settings.

### 3.5. Qualitative Study Results

To extend the quantitative results from the PLS-SEM analysis, a qualitative phase was conducted through in-depth interviews with physicians at community health centers. The aim was not simply to add on to the statistical findings, but to understand how those patterns actually play out in day-to-day clinical practice. Participants were asked to reflect on their experiences with EMR, particularly regarding patient interaction and teamwork. From the thematic analysis, three recurring themes began to take shape. These themes help clarify why digital health competency appears to be so influential, why data governance and information access show a more limited role, and how factors such as technostress and EMR mastery level shape system use.

A qualitative inquiry was conducted involving 12 physicians from community health centers. Participants were selected using a saturated sampling approach, in which interviews were continued until no substantially new themes or insights emerged from the data. The number of participants was considered adequate to achieve thematic saturation, particularly given the relatively focused scope of the study and the homogeneity of respondents’ professional backgrounds and EMR usage experiences.

To assure the qualitative findings, methodological triangulation was applied by comparing the interview narratives with the quantitative PLS-SEM results. The qualitative phase was designed to confirm statistical associations, and to provide contextual and practical insights regarding how EMR implementation influences patient interaction and interprofessional collaboration in daily clinical settings. In addition, investigator discussions were conducted during the coding process to ensure consistency in theme interpretation.

Due to manuscript length considerations and the complementary role of the qualitative phase within the mixed-method design, only three principal themes with the strongest explanatory relevance to the quantitative model are reported in this paper. Nevertheless, the selected themes adequately represent the dominant patterns identified across participants’ narratives.

**Theme 1**: Digital Health Competency as a Foundational Capability for Patient Engagement and Interprofessional Collaboration

The interviewees revealed a head of community health center opinion, “*As the head of a community health center, I see the digital health competency of healthcare workers as the most crucial factor because this ability directly influences how doctors and other healthcare professionals utilize EMR in their interactions with patients. If doctors possess strong digital competency, they can explain examination results, record patient data in real time, and involve patients in the clinical decision-making process. This leads to increased patient engagement*”. Furthermore, “*digital competency also facilitates coordination between healthcare professionals through the EMR system, thus enabling more effective interprofessional collaboration*.”

These statement highlights the central role of digital health competency in facilitating the effectiveness of EMR utilization in primary healthcare settings. Participants emphasized that healthcare workers with strong digital competencies are better able to leverage EMR features to support clinical communication with patients, document medical information efficiently, and involve patients in decision-making. These capabilities enhance patient engagement by allowing physicians to share information transparently and interactively during consultations. Additionally, digital competency was perceived as a key enabler of interprofessional collaboration, as proficient users can efficiently access and share patient information through the EMR system, thereby improving coordination among healthcare professionals within the primary care team.

**Theme 2:** Governance and System Infrastructure Are Perceived as Background Factors in Addition to Direct Drivers of Engagement and Collaboration

*“Data governance maturity and health information affordance (HIA) are often more structural and administrative in nature. At the service level in public health centers, healthcare workers are more influenced by their personal ability to use technology than by data governance policies, which are often not directly visible in clinical practice.”*

This statement suggests that data governance maturity and health information affordances are perceived by healthcare professionals as organizational or system-level structures that operate in the background of healthcare delivery. Although these elements are important for ensuring data security, system reliability, and regulatory compliance, they are not always directly experienced by physicians during routine clinical interactions. Instead, participants emphasized that individual capabilities, particularly the ability to use digital systems effectively, play a more immediate role in shaping how EMR systems support patient engagement and interprofessional collaboration. This perception helps explain why structural factors, such as data governance and information accessibility, may have only a limited direct influence on physicians’ everyday use of EMR systems in primary healthcare settings.

**Theme 3:** The Moderating Role of Technostress and EMR Mastery Level in EMR Utilization

A general practitioner explained that mastery of the EMR system greatly influences its effective use in daily clinical practice. According to the physician, “*when doctors are familiar with the system and understand its features well, documenting patient data becomes faster, and the system actually supports more efficient service delivery*”. However, the physician also noted that when the system is perceived as complex or when technical problems occur, healthcare workers may experience technostress, which can reduce their motivation to fully use the EMR. Similarly, the head of a community health center emphasized that healthcare workers with strong mastery of the EMR system tend to use it more consistently and confidently in patient care, whereas those who feel pressured by technological demands or lack sufficient training may experience difficulties that hinder optimal EMR use.

The interview findings indicate that technostress and EMR mastery level jointly influence the extent to which healthcare providers utilize EMR systems in primary healthcare settings. Participants highlighted that higher levels of EMR mastery enable physicians to use digital systems more efficiently and confidently in routine clinical practice. Conversely, technostress arising from system complexity, technical issues, or increased documentation workload may discourage healthcare providers from fully utilizing EMR functionalities. These findings suggest that the balance between technological pressure and users’ digital proficiency plays an important moderating role in shaping EMR utilization in primary healthcare services.

## 4. Discussion

This study demonstrates that EMR utilization appears to represent an important component of how digital technologies may support care delivery in primary healthcare settings. In the context of persistent patient safety challenges, including misdiagnosis and medication errors highlighted in primary care settings, the findings suggest that effective EMR use depends on how well digital systems are integrated into clinical workflows and enacted in everyday practice.

The results show that EMR utilization is shaped by the alignment between workflow processes, user capability, and system functionality, with direct implications for care coordination, patient engagement, and service efficiency. EMR utilization may be influenced not only by technical factors, but also by how digital systems support clinical coordination and routine decision-making processes in resource-constrained primary care settings [57,58].

The integration of quantitative and qualitative findings provides a more comprehensive understanding of how these factors operate in practice. While the structural model identifies digital health competency and clinical workflow fit as the strongest predictors of interprofessional collaboration and perceived patient engagement, the qualitative findings explain how these relationships are enacted in real-world clinical interactions. Clinicians with higher digital competency appeared more able to integrate EMR use into routine consultations and documentation processes, enabling real-time documentation, more effective communication, and can improved coordination of care [59,60].

The analysis indicates that competency enhances not only system usability but also the ability to deliver more coordinated, patient-centered services. The qualitative findings provided important contextual explanations for the quantitative relationships identified in the structural model. Participants consistently described digital health competency and workflow alignment as critical factors influencing effective EMR utilization in daily clinical practice, supporting the strong statistical associations observed in the quantitative phase.

A central finding of this quantitative–qualitative study is the role of clinical workflow fit as a key mechanism through which EMR systems contribute to care delivery. Besides acting as a direct predictor, workflow alignment operates through interprofessional collaboration and patient engagement, indicating that its impact is largely mediated by relational and process-based factors. When EMR systems align with clinical routines, they support smoother communication, reduce duplication, and enhance efficiency, thereby facilitating more consistent system use [12,13]. This alignment is particularly important for reducing the likelihood of clinical errors, as structured workflows and real-time access to information may help support more consistent clinical decision-making and reduce potential documentation-related risks.

Digital health competency was identified as an important factor associated with EMR utilization. Its strong direct and indirect effects suggest that competency is foundational to achieving meaningful system use in practice [14]. Clinicians with higher competency are better able to interpret, adapt, and apply digital tools within clinical workflows, thereby enhancing both patient engagement and interprofessional coordination. Similar patterns have been reported in recent studies from primary healthcare settings in Europe and Asia, where workflow integration and digital competency were associated with more effective EMR adoption and collaborative care practices [61,62]. Notably, the results emphasize that digital infrastructure initiatives may benefit from ongoing capacity-building efforts to ensure that technological potential translates into improved healthcare delivery.

In contrast, data governance maturity demonstrates a more selective role. While governance significantly influences perceived patient engagement, its lack of effect on interprofessional collaboration suggests that governance mechanisms primarily operate through process standardization and accountability rather than only communication enhancement [15]. Structured governance can improve reliability and data quality, thereby indirectly supporting service efficiency. However, excessive procedural rigidity may increase administrative burden and limit clinician engagement [16,17]. These findings suggest that governance should be designed to support clinical workflows instead of constraining them, particularly in resource-limited settings.

Health IT Affordances (HIAs) also play a conditional role in shaping EMR utilization. Although these capabilities represent the functional potential of digital systems, their impact depends on whether users are able to perceive and enact them in practice [19]. In primary healthcare environments characterized by high workload, advanced system features may remain underutilized if they are not aligned with workflow needs or user familiarity [63]. These results support previous studies suggesting that the effectiveness of digital systems depends partly on how they are incorporated into everyday clinical practice [23].

EMR mastery level further strengthens this interpretation. Its significant direct effect and moderating role indicate that advanced users are better able to translate collaborative processes into actual system use [12,16]. This suggests that system mastery enhances the effectiveness of communication-based processes in driving EMR utilization. However, its non-significant interaction with patient engagement indicates that certain performance-related aspects of system use may depend more on structural factors than individual capability. These findings are consistent with previous studies showing that higher levels of system mastery enable deeper and more meaningful engagement with digital health technologies [64,65].

Technostress introduces an important boundary condition in this context. While its direct effect suggests that system integration and technical support may facilitate utilization [17], its moderating effect indicates that excessive digital demands can weaken the positive influence of patient engagement on EMR use [18]. This reflects the cognitive and operational burdens clinicians experience when balancing patient care with system requirements. Consistent with prior research, technostress may reduce efficiency and discourage the use of more advanced system functionalities, thereby limiting the potential benefits of digital health systems [66,67]. Similarly, qualitative responses highlighted how technostress and varying levels of EMR mastery shaped clinicians’ ability to integrate EMR systems into routine workflows, thereby helping explain the moderating effects identified in the PLS-SEM analysis.

Not all hypothesized relationships were supported, and these findings provide important insights. The lack of effect of governance on collaboration and the limited role of affordance awareness suggest that structural or perceptual factors alone are insufficient to drive EMR use. Instead, utilization emerges when these conditions are translated into practical value within clinical workflows [22]. This highlights the importance of focusing on how digital systems support everyday clinical practice through their features.

From a theoretical perspective, this study extends existing views of digital health implementation by demonstrating that EMR utilization is shaped by the interaction between organizational alignment, user capability, and realized system potential [68,69]. In addition to treating system use as a direct outcome of technology acceptance, the findings show that the process towards EMR utilization is mediated through care processes such as communication, coordination, and patient engagement, which also aligns with a previous study [12].

From a practical standpoint, the findings highlight several priorities for improving primary healthcare delivery. Ensuring alignment between EMR systems and clinical workflows should be a key consideration in system design and implementation. Capacity-building initiatives should focus on developing applied digital competency and mastery of systems to support effective use in routine practice [23]. Governance frameworks should balance accountability with usability, while system development should prioritize features that directly enhance care coordination, patient interaction, and service efficiency [25]. These findings suggest that improving EMR utilization is not only essential for optimizing system performance, but also for strengthening patient safety by reducing variability in clinical practice and supporting more reliable and coordinated care processes.

This study also notes several limitations. Firstly, due to process improvements, which may not be static but can evolve at each community health center. This model should be tested in a longitudinal study to examine the sustainability of EMR utilization and its long-term impact on healthcare outcomes. Comparative studies across different healthcare settings may also provide insights into contextual variations in system use [26]. In addition, qualitative research could further explore how clinicians interpret and enact digital system capabilities in everyday clinical practice.

Overall, the proposed model indicates that EMR utilization should not be seen as a technical issue but a critical component of healthcare delivery, particularly in community health centers. The setting in one city may benefit from similar conditions and equal support and regulation for the community health centers. However, it may also limit the generalization to other cities within the country, which may have different rules. Therefore, it is necessary to create model tests through comparative studies with other cities.

The proposed model demonstrates adequate explanatory and predictive power, as indicated by the R^2^ and Q^2^ values, suggesting its robustness in explaining EMR utilization processes and their potential benefits for patient care. The findings further indicate that meaningful system use emerges when digital technologies are effectively integrated into clinical workflows, supported by user capability, and aligned with the realities of primary care practice [27]. Strengthening these factors may contribute to improved care coordination, patient engagement, and service efficiency in primary healthcare settings.

## 5. Conclusions

This study developed and empirically examined an integrated model of EMR utilization in Indonesian primary healthcare settings to better understand the socio-technical factors influencing digital health implementation. The findings demonstrate that EMR utilization is shaped not only by technological availability but also by the interaction among clinical workflow alignment, digital health competency, organizational conditions, and user-related factors.

The results identified clinical workflow fit and digital health competency as the most influential antecedents of EMR utilization, primarily through their positive relationships with interprofessional collaboration and perceived patient engagement. These findings suggest that effective EMR utilization is more likely to occur when digital systems are aligned with routine clinical practice and supported by adequate user capability. The qualitative findings further reinforced the importance of workflow integration, practical usability, and professional adaptation in supporting sustainable EMR use in primary healthcare settings.

In contrast, some organizational and contextual factors demonstrated more selective or conditional effects. Data governance maturity contributed primarily to process-related support mechanisms rather than directly influencing all dimensions of EMR utilization. Similarly, technostress and EMR mastery level functioned as moderating factors that influenced how healthcare professionals interacted with EMR systems in daily practice. These findings indicate that digital transformation in primary healthcare requires careful balancing between governance structures, usability, and workload demands.

Overall, this study contributes to the growing literature on digital health implementation in resource-constrained primary healthcare settings by demonstrating that meaningful EMR utilization depends on the alignment between technology, clinical workflows, and human capability. From a practical perspective, sustainable EMR implementation should prioritize workflow integration, competency development, and supportive organizational environments to strengthen care coordination, patient engagement, and the quality and safety of healthcare delivery.

## Figures and Tables

**Table 1:** Construct Definition.

Constructs	Conceptualized Definition	References
Health IT Affordances	Health IT affordances refer to the capabilities that emerge from the interaction between health information technologies and users, enabling the effective performance of clinical, administrative, and communication tasks within healthcare workflows.	[31,32]
Data Governance Maturity	Data governance maturity is defined as the level of organizational capability in managing data quality, security, standardization, accessibility, and regulatory compliance through structured policies, processes, and oversight mechanisms.	[33]
Clinical Workflow Fit	Clinical workflow fit refers to the degree to which an EMR system aligns with and supports existing clinical processes, routines, and task sequences without causing disruption or inefficiency.	[21]
Digital Health Competency	Digital health competency is the ability of healthcare professionals to effectively use digital technologies, including EMR systems, for clinical decision-making, documentation, communication, and patient management.	[34]
Perceived Patient Engagement	Perceived patient engagement refers to healthcare workers’ belief that digital systems can enhance patient involvement, communication, shared decision-making, and participation in their own care.	[35]
Interprofessional Collaboration Enabled	Interprofessional collaboration is defined as the extent to which electronic medical record systems facilitate communication, information sharing, coordination, and teamwork among different healthcare professionals.	[16]
EMR Mastery Level	EMR mastery level refers to the degree of proficiency, confidence, and efficiency demonstrated by healthcare professionals in operating and utilizing EMR system features in clinical practice.	[36]
Technostress	Technostress is the psychological strain experienced by healthcare professionals due to the demands of using digital technologies, including overload, complexity, uncertainty, and perceived technological pressure.	[18]
EMR Utilization	EMR utilization refers to the extent and frequency with which healthcare professionals actively use electronic medical record systems in their daily clinical documentation, decision-making, and patient care activities.	[37]

**Table 2:** Respondent profile (*n* = 217).

Characteristics	Category	*n*	%
Active Medical License (STR)	Yes	217	100.0
	No	0	0.0
Employment Status	Civil Servant	112	51.6
	Temporary Contract	54	24.9
	Others	51	23.5
Experience Using EMR	Yes	217	100.0
	No	0	0.0
Sex	Male	57	26.7
	Female	159	73.3
Age	20–30 years	77	35.5
	31–40 years	95	43.8
	41–50 years	40	18.4
	>50 years	5	2.3
Highest Education Level	Bachelor of Medicine/Professional Degree	150	69.1
	Master’s Degree	40	18.4
	Specialist	18	8.3
	Others	9	4.1
Years of Working at Primary Health Center	<1 year	54	24.9
	1–3 years	56	25.8
	4–6 years	45	20.7
	7–10 years	27	12.4
	>10 years	35	16.1
Number of Health Centers Worked In	1 Health Center	112	51.6
	>1 Health Center	105	48.4
Frequency of EMR Use	Never	0	0
	Rarely	30	13.8
	Frequently	187	86.2
EMR Training	Yes	101	46.5
	No	116	53.5

**Table 3:** Reliability and convergent validity.

Variable	Code	Indicator	Mean	OL
Clinical Workflow Fit	CWF1	Data entry for the EMR aligns with the patient examination process.	4.290	0.828
	CWF2	Data entry in the EMR can be effectively implemented in emergency case management at the Community Health Center.	4.230	0.770
	CWF3	The EMR features align with my work requirements in patient care at the outpatient at the Community Health Center.	4.341	0.795
CA = 0.715, rho_a = 0.721, rho_c = 0.840, AVE = 0.637				
Data Governance Maturity	DGM1	The Community Health Center has clear Standard Operating Procedures for filling in EMR data.	4.364	0.765
	DGM2	The SOPs implemented at the Community Health Center indicate a rule that data may not be disseminated without permission.	4.281	0.756
	DGM3	The SOPs implemented at the Community Health Center cover data access security by EMR users.	4.249	0.776
	DGM4	The SOPs shows the sequence of filling in EMR	4.240	0.810
CA = 0.781, rho_a = 0.781, rho_c = 0.859, AVE = 0.604				
Digital Health Competency	DHC1	I am able to accurately record clinical data in the EMR.	4.364	0.739
	DHC2	I understand how the EMR system works in patient care.	4.157	0.624
	DHC3	I can use EMR features to support diagnostic/therapeutic decision making.	4.373	0.739
	DHC4	I am able to solve basic problems that arise in using EMR.	4.359	0.768
CA = 0.690, rho_a = 0.704, rho_c = 0.810, AVE = 0.518				
EMR Mastery Level	EML1	I have mastered the use of EMR in the entire patient care process.	4.309	0.758
	EML2	I know and understand the important features available in EMR.	4.323	0.733
	EML3	I am able to assist other healthcare workers in using EMR.	4.240	0.792
	EML4	I am able to learn and adapt to new EMR features quickly.	4.332	0.741
	EML5	I feel I have a high level of competence in using EMR.	4.263	0.806
CA = 0.825, rho_a = 0.829, rho_c = 0.877, AVE = 0.588				
EMR Utilization	EMU1	EMR reduces the potential for service errors at this health center.	4.327	0.755
	EMU2	EMR supports faster and more accurate clinical decision-making in Community Health Centers.	4.290	0.742
	EMU3	The use of EMR helps me in establishing the correct clinical diagnosis by providing complete, relevant, and timely patient information during the service process at Community Health Centre.	4.373	0.756
	EMU4	The quality of outpatient services at the Community Health Center has improved since EMR has been used routinely.	4.392	0.785
	EMU5	The EMR feature helps improve patient safety during the service process at the Community Health Center.	4.406	0.755
CA = 0.816, rho_a = 0.816, rho_c = 0.872, AVE = 0.576				
Health IT Affordances	HIA1	Information technology makes it easier for me to coordinate with other colleagues.	4.327	0.867
	HIA2	Information technology can support the clinical diagnosis.	4.240	0.883
	HIA3	Information technology can help provide fast information regarding a patient’s medical history.	4.401	0.534
	HIA4	Information technology can help speed up the patient service workflow at Community Health Centers.	4.267	0.909
	HIA5	Information technology can help me prevent medical errors such as prescribing the wrong medication to patients with allergies.	4.304	0.516
CA = 0.795, rho_a = 0.801, rho_c = 0.868, AVE = 0.581				
Inter-professional Collaboration Enabled	ICE1	In my experience, the use of EMR makes communication between healthcare professionals at the Community Health Center easier.	4.332	0.760
	ICE2	The use of EMR improves coordination of services between units at the Community Health Center.	4.309	0.794
	ICE3	Patient information becomes easier to share between healthcare professionals via EMR.	4.382	0.708
	ICE4	The use of EMR makes it easier to divide tasks between health workers in patient care.	4.387	0.708
CA = 0.729, rho_a = 0.730, rho_c = 0.831, AVE = 0.552				
Perceived Patient Engagement	PPE1	Based on my experience, the use of EMR increases patient involvement in the care process.	4.240	0.728
	PPE2	The patients I serve become more active in understanding their health conditions through EMR.	4.180	0.823
	PPE3	EMR helps the patients I serve to follow their established care plans.	4.253	0.785
	PPE4	The use of EMR improves the quality of communication between patients and healthcare professionals.	4.194	0.781
CA = 0.787, rho_a = 0.796, rho_c = 0.861, AVE = 0.608				
Techno stress	TCS1	I feel that the use of digital systems/EMR at work increases my workload.	4.359	0.739
	TCS2	I find it difficult to understand the new features in the technology systems used in the Community Health Center.	4.406	0.864
	TCS3	I feel I must work faster because of the demands of using technology in patient care.	4.295	0.816
CA = 0.734, rho_a = 0.734, rho_c = 0.849, AVE = 0.653				

CA = Cronbach’s alpha, OL = outer loading, AVE = average variance extracted.

**Table 4:** Heterotrait–monotrait ratio.

Variable	CWF	DGM	DHC	EML	EMU	HIA	ICE	PPE	TCS	EML-PPE	EML-ICE	TCS-PPE	TCS-ICE
CWF													
DGM	0.585												
DHC	0.880	0.600											
EML	0.664	0.460	0.948										
EMU	0.901	0.626	0.964	0.855									
HIA	0.764	0.487	0.728	0.623	0.803								
ICE	0.881	0.567	0.910	0.818	0.888	0.682							
PPE	0.766	0.533	0.842	0.724	0.833	0.569	0.720						
TCS	0.646	0.405	0.666	0.627	0.714	0.519	0.712	0.509					
EML × PPE	0.415	0.244	0.390	0.455	0.392	0.236	0.457	0.325	0.258				
EML × ICE	0.390	0.274	0.313	0.341	0.305	0.168	0.414	0.402	0.261	0.857			
TCS × PPE	0.385	0.339	0.339	0.326	0.414	0.230	0.391	0.271	0.272	0.733	0.693		
TCS × ICE	0.363	0.339	0.342	0.308	0.386	0.203	0.374	0.325	0.365	0.615	0.725	0.742	

CWF = clinical workflow fit, DGM = data governance maturity, DHC = digital health competency, EML = EMR mastery level, EMU = EMR utilization, HIA = Health IT affordances, ICE = interprofessional collaboration enabled, PPE = perceived patient engagement, TCS = technostress.

**Table 5:** CVPAT results.

Variable/Model	PLS-SEM vs. Indicator Average (IA)				PLS-SEM vs. Linear Model (LM)			
	PLS Loss	IA Loss	Average Loss Difference	*p*-Value	PLS Loss	LM Loss	Average Loss Difference	*p*-Value
Interprofessional Collaboration Enabled	0.356	0.493	−0.137	0.000	0.356	0.375	−0.019	0.136
Perceived Patient Engagement	0.459	0.627	−0.168	0.000	0.459	0.507	−0.049	0.001
EMR Utilization	0.292	0.470	−0.179	0.000	0.292	0.317	−0.025	0.031
Overall	0.363	0.525	−0.162	0.000	0.363	0.394	−0.031	0.000

**Table 6:** Hypothesis testing results.

Hypotheses		Coefficient (*β*)	SD	*p*-Values	Confidence Interval		Result
					5.0%	95.0%	
H1	Health IT Affordances -> Interprofessional Collaboration Enabled	0.108	0.062	0.040	0.009	0.211	Hypothesis Supported
H2	Health IT Affordances -> Perceived Patient Engagement	0.048	0.069	0.244	−0.062	0.165	Hypothesis not supported
H3	Data Governance Maturity -> Interprofessional Collaboration Enabled	0.085	0.065	0.097	−0.021	0.192	Hypothesis not supported
H4	Data Governance Maturity -> Perceived Patient Engagement	0.112	0.062	0.035	0.015	0.218	Hypothesis Supported
H5	Clinical Workflow Fit -> Interprofessional Collaboration Enabled	0.304	0.086	0.000	0.160	0.443	Hypothesis Supported
H6	Clinical Workflow Fit -> Perceived Patient Engagement	0.276	0.080	0.000	0.137	0.399	Hypothesis Supported
H7	Digital Health Competency -> Interprofessional Collaboration Enabled	0.377	0.065	0.000	0.272	0.484	Hypothesis Supported
H8	Digital Health Competency -> Perceived Patient Engagement	0.388	0.070	0.000	0.277	0.504	Hypothesis Supported
H9	Perceived Patient Engagement -> EMR Utilization	0.370	0.067	0.000	0.257	0.476	Hypothesis Supported
H10	Interprofessional Collaboration Enabled -> EMR Utilization	0.228	0.059	0.000	0.132	0.324	Hypothesis Supported
H11	EMR Mastery Level × Interprofessional Collaboration Enabled -> EMR Utilization	0.224	0.089	0.006	0.063	0.354	Hypothesis Supported
H12	EMR Mastery Level × Perceived Patient Engagement -> EMR Utilization	−0.098	0.070	0.080	−0.194	0.036	Hypothesis not supported
H13	Technostress × Interprofessional Collaboration Enabled -> EMR Utilization	−0.077	0.073	0.144	−0.188	0.051	Hypothesis not supported
H14	Technostress × Perceived Patient Engagement -> EMR Utilization	−0.139	0.070	0.023	−0.248	−0.018	Hypothesis supported

SD = Standard deviation.

**Table 7:** Mediation results.

Path	Coefficient Indirect	*p*-Value Indirect	*p*-Value Direct Effect	Interpretation
HIA -> PPE -> EMU	0.018	0.249	0.020	No effect-no mediation
HIA -> ICE -> EMU	0.025	0.072		No effect-no mediation
CWF -> PPE -> EMU	0.102	0.003	0.000	Partial Mediation
CWF -> ICE -> EMU	0.069	0.006		Partial Mediation
DGM -> PPE -> EMU	0.041	0.037	0.023	Partial Mediation
DGM -> ICE -> EMU	0.019	0.125		Direct only
DHC -> PPE -> EMU	0.144	0.000	0.000	Partial Mediation
DHC -> ICE -> EMU	0.086	0.000		Partial Mediation

CWF = clinical workflow fit, DGM = data governance maturity, DHC = digital health competency, EMU = EMR utilization, HIA = Health IT affordances, ICE = interprofessional collaboration enabled, PPE = perceived patient engagement.

## Data Availability

The datasets generated and/or analyzed during the current study are not publicly available due to ethical and confidentiality considerations involving healthcare professionals and institutional data from community health centers.
